# Monitoring of serum magnesium levels during long-term use of proton pump inhibitors in elderly japanese patients: is it really necessary or not?

**DOI:** 10.1186/s40780-022-00266-7

**Published:** 2022-12-14

**Authors:** Nobuhiko Nakamura, Kanaha Yoneda, Takashi Higo, Masaaki Kusumoto

**Affiliations:** 1 Education and Research Center for Clinical Pharmacy, Kyoto Pharmaceutical University, 5-Nakauchi-cho, Misasagi, Yamashina-ku, 607-8414 Kyoto, Japan; 2 Takashi Higo, Higo Internal Medicine Clinic, 813-Hama, 625-0036 Maizuru-city, Kyoto, Japan; 3 Masaaki Kusumoto, Ai Pharmacy, 801-Hama, 625-0036 Maizuru-city, Kyoto, Japan

**Keywords:** Proton pump inhibitors, Long-term use, Serum magnesium, Hypomagnesemia, Elderly patients, Japan

## Abstract

**Background:**

Long-term use of proton pump inhibitors (PPIs) has been found to significantly lower serum magnesium levels in patients in the USA and Europe. The package inserts of PPIs in these countries clearly state that healthcare professionals should consider monitoring magnesium levels prior to initiation of PPI treatment and periodically thereafter. However, the package inserts of PPIs in Japan do not clearly mention the monitoring of magnesium levels. In this study, we evaluated the relationship between long-term use of PPIs and the lower serum magnesium concentrations in elderly Japanese patients.

**Methods:**

Using a retrospective observational approach, a total of 264 Japanese outpatients were included in the study. Patients over the age of 75 years were considered elderly. Serum magnesium levels of the patients were measured in units of 0.1 mg/dL between January 2016 and June 2022 at the Higo Internal Medicine Clinic and Ai Pharmacy in Kyoto, Japan.

**Results:**

Four of the 264 eligible patients were diagnosed with hypomagnesemia. Three were PPI non-users, and one was a PPI user. Serum magnesium concentrations were significantly lower in PPI users (*n* = 47) than in non-users (*n* = 85; 2.1 ± 0.2 vs. 2.2 ± 0.3 mg/dL, *p* < 0.05) in the 132 elderly patients. Comorbidity included diabetes mellitus in both PPI users (23.4%) and non-users (57.6%) and hyperlipidemia in both PPI users (61.7%) and non-users (41.2%).

**Conclusion:**

PPIs are commonly used oral drugs for elderly patients. There was an association between the long-term use of PPIs and lower serum magnesium concentrations in elderly patients. Although the difference in the decrease in serum magnesium concentrations was within the normal range of serum magnesium levels, health care professionals should consider monitoring serum magnesium levels periodically in elderly patients receiving long-term PPIs.

## Background

Long-term use of proton pump inhibitors (PPIs) has been found to significantly lower serum magnesium levels in the USA and Europe outpatients and inpatients [1, 2]. In 2011, the US Food and Drug Administration (FDA) issued a safety information stating that long-term use of PPIs may result in hypomagnesemia [3]. The package inserts of PPIs in the USA and Europe clearly state the following: ‘For patients expected to be on prolonged treatment or who take PPIs with medications such as digoxin or drugs that may cause hypomagnesemia (e.g., diuretics), health care professionals may consider monitoring magnesium levels prior to initiation of PPI treatment and periodically’ [4]. However, the PPI package inserts in Japan do not clearly mention about magnesium level monitoring.

PPIs are commonly used as oral drugs for treating gastroesophageal reflux disease [5] and nonsteroidal anti-inflammatory drug-associated gastric ulcers [6] in elderly patients. In the West, the effects of long-term use of PPIs are perceived in one out of nine individuals in the elderly population (median age, 74 years), and in four out of ten, no reason for PPI use can be identified [7]. In Japan, PPIs have been used in 11,981 (36.9%) patients to prevent gastrointestinal bleeding events among elderly (aged ≥ 75 years) Japanese patients with non-valvular atrial fibrillation [8]. In Kyoto Prefecture, Japan, the percentage of population aged ≥ 75 years was 15.3% in 2021. Yet, no study has been conducted till date on the long-term use of PPIs in elderly Japanese patients.

Long-term use of PPIs has been found to significantly lower serum magnesium levels in patients. However, the study population included patients with cirrhosis receiving long-term PPIs [9] or those undergoing hemodialysis [10]. Patients with cirrhosis and hemodialysis, receiving long-term PPI treatment, are rare in regional clinics. Overall, attention to common chronic diseases, such as hypertension or diabetes, in elderly patients receiving long-term PPI treatment is lacking. Furthermore, whether physicians in regional clinics should consider monitoring magnesium levels periodically and prior to the initiation of PPI treatment, remains to be clarified. Therefore, we evaluated the relationship between long-term use of PPIs and lower serum magnesium concentrations in elderly Japanese outpatients aged ≥ 75 years.

## Methods

### Study design and patients

The current retrospective observational study was conducted at the Higo Internal Medicine Clinic and Ai Pharmacy in Kyoto. The Higo Internal Medicine Clinic has a primary care practice that focuses on digestive and cardiovascular diseases. The Ai Pharmacy collaborates with clinics so that pharmacists can provide continuous healthcare support to patients. We studied the serum magnesium levels in units of 0.1 mg/dL for 344 outpatients in the clinic between January 2016 and June 2022. Serum magnesium levels were collected from the medical records in the clinic. Data on PPI users and non-users were collected from the prescribed pharmacy records. Data from medical and prescription records were matched by the university. The inclusion criteria were as follows: (i) age > 20 years and (ii) blood sampling after at least a 10-h overnight fast. The exclusion criteria were as follows: (i) patients who were administered magnesium [11], (ii) patients who were administered diuretics [12], and (iii) patients with confirmed malignancy [13].

Patients were stratified into PPI users and non-users. Patients who were administered a PPI for > 6 months were regarded as PPI users, whereas those not administered a PPI at all were considered PPI non-users. A previous study reported that the time that elapsed between the start of PPI use and first clinical detection of PPI-induced hypomagnesemia ranged from 2 weeks to 13 years [14]. In a prospective open-label comparative study, stable serum magnesium levels were observed after 12 months and found no association between PPI use and risk of hypomagnesemia in the general population [15]. On the other hand, another study reported that the risk of hypomagnesemia was increased with prolonged use of PPI (> 6 months) in the general population [12]. Therefore, in this study, long-term PPI use was defined as > 6 months, which has shown to increase the risk of hypomagnesemia. Patients were prescribed PPI therapy at the dosage level approved in Japan (omeprazole or esomeprazole 20 mg, lansoprazole 30 mg, or rabeprazole 10 mg or 20 mg once daily).

### Assessments

First, we evaluated the relationship between long-term use of PPIs and lower serum magnesium concentrations in elderly patients. Elderly patients were defined as those aged ≥ 75 years, in compliance with previous studies in Japan [8], and from the perspective of medical insurance for the elderly under Japan’s universal health care system. Hypomagnesemia was defined as a plasma magnesium concentration less than 1.8 mg/dL [16, 17]. Basic information regarding patient characteristics, including sex, age, laboratory data, and comorbidity, were collected from medical records and prescriptions. Comorbidity were reviewed to determine chronic diseases, such as diabetes mellitus, hypertension, hyperlipidemia, liver disease, renal disease, allergic disease, and respiratory disease.

### Statistical analysis

PPI users and non-users were compared using Fisher’s exact test or Mann–Whitney U test to assess serum magnesium levels and patients’ clinical characteristics, as appropriate. No imputation was made for missing data that were not included in the analyses. Data were analyzed using Bell Curve for Excel 3.21 (Social Survey Research Information, Tokyo, Japan). Statistical significance was set with a *p*-value < 0.05.

## Results

**Fig. 1:**
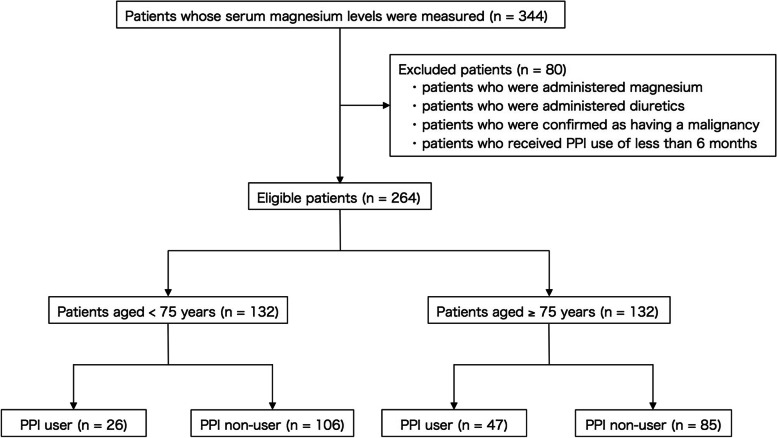
Flowchart of the study

As shown in Fig. 1, serum magnesium levels were measured for 344 patients, of whom 80 were ineligible, including those who were administered magnesium oxide and diuretics. After excluding the 80 ineligible patients, 264 patients were finally analyzed. Of the 264, 132 were aged ≥ 75 years, and 132 were aged < 75 years. There was no patient with cirrhosis or hemodialysis among the 264 patients.

**Fig. 2:**
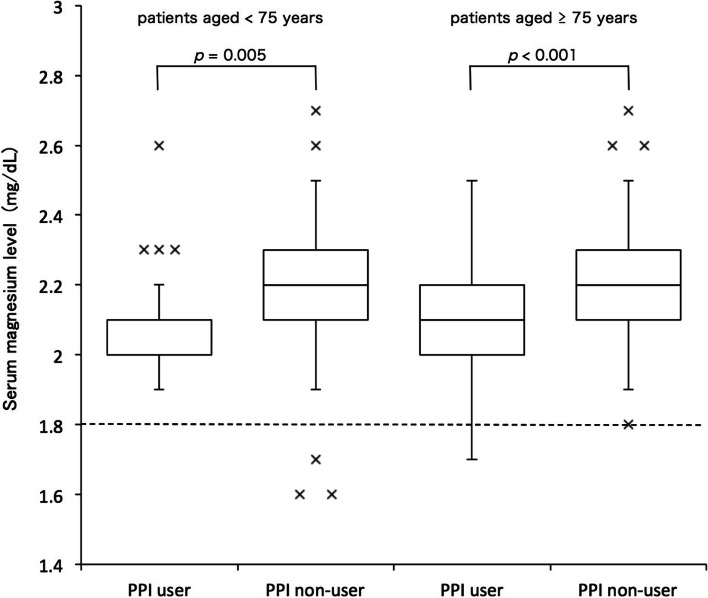
Serum magnesium levels in proton pump inhibitor (PPI) users and non-users between patients aged < 75 years and those aged ≥ 75 years. For patients aged < 75 years, PPI users, *n* = 26; PPI non-users, *n* = 106. For patients aged ≥ 75 years, PPI users, *n* = 47; PPI non-users, *n* = 85. Data were analyzed by two-tailed Mann–Whitney U-test. The boxes indicate the third quartile (Q3) at the top of the box, median, and first quartile (Q1) at the bottom. The whiskers show 5 percentiles at the bottom end and 95 percentiles at the top end. Cross symbols are considered to be outliers. IQR: interquartile range. The horizontal dotted line indicates the lower limit of reference values for serum magnesium levels, i.e., 1.8 mg/dL. Four of the 264 eligible patients were diagnosed with hypomagnesemia

Table 1 shows the characteristics of patients aged ≥ 75 years. Of the 132 patients aged ≥ 75 years, 47 were PPI users, and 85 were non-users. There was no significant difference in the sex ratio (male/female) (*p* = 0.460). Their mean ages were 83.7 ± 5.5 in PPI users and 81.4 ± 5.2 years in PPI non-users (*p* = 0.005). Serum magnesium levels in PPI users and non-users between patients aged < 75 years and ≥ 75 years are shown in Fig. 2. One patient among the PPI users showed symptoms of hypomagnesemia, as the plasma magnesium concentration was < 1.8 mg/dL. Serum magnesium concentrations were significantly lower in PPI users (*n* = 47) than in non-users (*n* = 85; 2.1 ± 0.2 vs. 2.2 ± 0.3 mg/dL, *p* < 0.05) (Table 1). Comorbidity included diabetes mellitus in both PPI users (23.4%) and non-users (57.6%) (*p* < 0.001) and hyperlipidemia in both PPI users (61.7%) and non-users (41.2%) (*p* < 0.05). There was no significant difference between PPI users and non-users in the occurrence of hypertension, liver disease, renal disease, allergic disease, or respiratory disease. Serum magnesium concentrations were similar in patients taking the PPI esomeprazole (2.2 ± 0.17 mg/dL) or lansoprazole (2.1 ± 0.18 mg/dL, *p* = 0.130) (Table 2).

**Table 1:** Patients' clinical characteristics (aged ≥ 75 years)

	PPI (+)	PPI (-)	***P*** value
Gender male/female, (%)	16/31 (34.0/66.0)	35/50 (41.2/58.8)	0.460
Age mean, years	83.7 ± 5.5	81.4 ± 5.2	0.013
Laboratory measurements			
Magnesium (mg/dL)	2.1 ± 0.2	2.2 ± 0.3	0.005
Sodium (mEq/dL)	141.2 ± 2.9	140.9 ± 2.4	0.275
Potassium (mEq/dL)	4.1 ± 0.4	4.3 ± 0.5	0.011
Blood urea nitrogen (mg/dL)	17.6 ± 5.0	20.3 ± 9.3	0.176
eGFR (mL/min)	62.5 ± 17.1	59.2 ± 19.0	0.484
Creatinine (mg/dL)	0.8 ± 0.3	0.9 ± 0.5	0.442
Albumin (g/dL)	4.1 ± 0.3	4.1 ± 0.4	0.549
Disorder present/absent, (%)			
Diabetes mellitus	11/36 (23.4/76.6)	49/36 (57.6/42.4)	<0.001
Hypertension	36/11 (76.6/23.4)	58/27 (68.2/31.8)	0.422
Hyperlipidemia	29/18 (61.7/38.3)	35/50 (41.2/58.8)	0.030
Liver disease	2/45 (4.3/95.7)	2/83 (2.4/97.6)	0.616
Renal disease	3/44 (6.4/93.6)	10/75 (11.8/88.2)	0.378
Allergic disease	3/44 (6.4/93.6)	5/80 (5.9/94.1)	1.000
Respiratory disease	0/47 (0.0/100.0)	3/82 (3.5/96.5)	0.552

*Abbreviation*: *eGFR* Estimated glomerular filtration rate

**Table 2:** Serum magnesium concentrations in patients receiving two types of PPIs (aged ≥ 75 years)

	Esomeprazole (*n* = 11)	Lansoprazole (*n* = 36)	*P* value
Magnesium (mg/dL), mean ± SD (range)	2.2 ± 0.17 (1.90–2.50)	2.1 ± 0.18 (1.70–2.50)	0.130

*Abbreviation*: *PPI* Proton pump inhibitor

Table 3 shows the characteristics of patients aged < 75 years. Twenty-four out of 132 patients were PPI users, and 106 were non-users. There was no significant difference in the sex ratio (male/female) (*p* = 0.358) (Table 3). Their mean ages were 64.4 ± 9.5 in PPI users and 65.5 ± 9.8 years in PPI non-users, respectively (*p* = 0.553). In patients aged < 75 years, serum magnesium concentrations were significantly lower in PPI users (*n* = 26) than in non-users (*n* = 106; 2.1 ± 0.1 vs. 2.2 ± 0.2 mg/dL, *p* < 0.001). Three patients among the PPI non-users showed symptoms of hypomagnesemia, as their plasma magnesium concentrations were < 1.8 mg/dL (Fig. 2). Three patients did not show hypomagnesemia symptoms, thus their treatment was maintained. Hypomagnesemia was not observed in PPI users. There were significant differences in blood urea nitrogen levels between PPI users (14.1 ± 3.0 mg/dL) and non-users (15.9 ± 3.8 mg/dL) in patients aged < 75 years. There was no significant difference in other laboratory data between PPI users and non-users. There was no significant difference in the occurrence of any comorbidity as well between PPI users and non-users. Serum magnesium concentrations were similar in patients taking the PPI esomeprazole (2.2 ± 0.11 mg/dL) or lansoprazole (2.1 ± 0.15 mg/dL, *p* = 0.087) (Table 4).

**Table 3:** Patients' clinical characteristics (aged < 75 years)

	PPI (+)	PPI (-)	***P*** value
Gender male/female, (%)	16/10 (61.5/38.5)	54/52 (50.9/49.1)	0.385
Age mean, years	64.4 ± 9.5	65.5 ± 9.8	0.553
Laboratory measurements			
Magnesium (mg/dL)	2.1 ± 0.1	2.2 ± 0.2	<0.001
Sodium (mEq/dL)	140.9 ± 3.1	141.2 ± 1.8	0.829
Potassium (mEq/dL)	4.2 ± 0.3	4.3 ± 0.4	0.419
Blood urea nitrogen (mg/dL)	14.1 ± 3.0	15.9 ± 3.8	0.023
eGFR (mL/min)	73.6 ± 13.0	70.2 ± 23.8	0.096
Creatinine (mg/dL)	0.8 ± 0.2	0.8 ± 0.3	0.501
Albumin (g/dL)	4.3 ± 0.3	4.3 ± 0.3	0.745
Disorder present/absent, (%)			
Diabetes mellitus	12/14 (46.2/53.8)	67/39 (63.2/36.8)	0.124
Hypertension	18/8 (69.2/30.8)	72/34 (67.9/32.1)	1.000
Hyperlipidemia	13/13 (50.0/50.0)	54/52 (50.9/49.1)	1.000
Liver disease	0/26 (0.0/100.0)	6/100 (5.7/94.3)	0.598
Renal disease	0/26 (0.0/100.0)	7/99 (6.6/93.4)	0.344
Allergic disease	0/26 (0.0/100.0)	7/99 (6.6/93.4)	0.344
Respiratory disease	0/26 (0.0/100.0)	4/102 (3.8/96.2)	0.585

*Abbreviation*: *eGFR* Estimated glomerular filtration rate

**Table 4:** Serum magnesium concentrations in patients receiving two types of PPIs (aged < 75 years)

	Esomeprazole (*n* = 6)	Lansoprazole (*n* = 20)	*P* value
Magnesium (mg/dL), mean ± SD (range)	2.2 ± 0.11 (2.00–2.30)	2.1 ± 0.15 (1.90–2.60)	0.087

*Abbreviation*: *PPI *Proton pump inhibitor

## Discussion

We evaluated the relationship between long-term use of PPIs and lower serum magnesium concentrations. Four patients, among the 264 analyzed, had hypomagnesemia. Hypomagnesemia is defined as a plasma magnesium concentration < 1.8 mg/dL [16, 17], and may cause serious conditions, such as tetany, spasms, and arrhythmias [9]. Symptoms of hypomagnesemia were not observed in the four patients; mild hypomagnesemia may be asymptomatic. Of the four patients, three were PPI non-users, and one was PPI user. There was a relationship between long-term use of PPIs and lower serum magnesium concentrations in this study. Our result is consistent with that of several previous reports [1, 2]. However, the difference in the decrease in their serum magnesium concentrations was small.

Nevertheless, there was a relationship between long-term use of PPIs and lower serum magnesium concentrations in the 132 elderly patients, aged ≥ 75 years, in this study. One patient aged ≥ 75 years had hypomagnesemia (i.e., plasma magnesium concentration < 1.8 mg/dL). One patient was a female in her 90s and was using PPI. Symptoms of hypomagnesemia were not observed, and the patient was taking the medications. Our results, however, were consistent with those of previous reports on the USA and Europe [2] and Japanese outpatients [9]. A previous study had reported Japanese patients with cirrhosis [9], who had serum magnesium levels lower than those without cirrhosis [9]. In addition, serum magnesium levels in PPI-using patients undergoing hemodialysis were found to be lower than those in patients receiving histamine 2 receptor antagonists [10]. However, in patients undergoing hemodialysis, the mean serum magnesium level of the subjects was 2.52 mg/dL among PPI users. Serum magnesium concentrations were higher in patients undergoing hemodialysis than in PPI users (2.1 ± 0.2 mg/dL) and non-users (2.2 ± 0.3 mg/dL) in this study. Here, eligible patients did not include patients with cirrhosis or hemodialysis, and rather included those with diabetes mellitus and hypertension. Serum magnesium concentrations have been implicated in diabetes mellitus [18] and hypertension [19]. Therefore, the results indicated comorbidities as vital factors that affect serum magnesium concentrations.

In contrast, in the 132 patients aged < 75 years, serum magnesium concentrations were significantly lower in PPI users (*n* = 26) than in non-users. Three patients had hypomagnesemia, with a plasma magnesium concentration < 1.8 mg/dL; both were PPI non-users. Two patients were male, one in his 20s and the other in his 60s. While the former was taking antithyroid medications, the latter had liver disease. One female patient in her 70s had comorbidities, such as diabetes mellitus, hypertension, and hyperlipidemia. In addition, there was no significant difference between PPI users and non-users regarding the occurrence of any comorbidity.

Serum magnesium levels did not differ across patients taking the two types of PPIs (esomeprazole and lansoprazole) (Tables 2 and 4), regardless of age; our results were consistent with previous studies [9, 10]. Therefore, PPI therapies should focus on symptoms of hypomagnesemia that cause serious conditions, such as tetany, spasms, and arrhythmias, regardless of the patient’s age or PPI type. Severe hypomagnesemia (0.4 mg/dL) has been reported to improve remarkably within a week of magnesium supplementation [20]. Therefore, for patients showing signs of lower serum magnesium concentrations, medical staff, such as physicians and pharmacists, should follow up promptly.

The mechanism of PPI-induced reduction in magnesium concentrations remains undetermined. Renal magnesium handling is normal in patients with PPI-induced hypomagnesaemia; the mechanism of PPI-induced decrease in magnesium concentration may be related to the gastrointestinal system [21]. Magnesium transport from the lumen into the epithelial cells is mediated by the transport proteins transient receptor potential melastatin 6/7 (TRPM6/7) [22]. Current evidence shows that carriers of TRPM6/7 mutations [23] and SNPs in TRPM6 could be at risk of developing hypomagnesemia during chronic PPI use [24].

The current study has several limitations. First, the possibility that patients may have visited multiple clinics and pharmacies is undeniable, although primary care practice was performed at a single clinic and pharmacy. Second, we were unable to study the course of serum magnesium levels after the initiation of PPI treatment. Third, although we were able to evaluate the relationship between long-term use of PPIs and lower serum magnesium concentrations, evidence for other side effects was unavailable. Fourth, the nutritional status that could affect serum magnesium levels was unknown. Fifth, in this study, serum calcium levels were not monitored. PPI-induced hypomagnesemia is reported to be associated with hypocalcemia and hypopotassemia [15, 23]. If symptoms of hypomagnesemia are observed, levels of electrolytes, such as calcium and potassium, should also be monitored. Notably, PPIs are not available over-the-counter in Japan. More data is needed to assess if it contradicts with previous reports in the USA and Europe. Therefore, further analysis on the long-term use of PPIs in elderly Japanese patients is needed.

## Conclusion

PPIs are commonly used as oral drugs in elderly patients. There was an association between the long-term use of PPIs and lower serum magnesium concentrations in elderly patients aged ≥ 75 years. Although the difference in the decrease in their serum magnesium concentrations was within the normal range of serum magnesium levels, health care professionals should consider monitoring serum magnesium levels periodically in elderly patients receiving long-term PPIs.

## Data Availability

All data generated or analyzed during this study are included in this published article.

## Abbreviations

FDA: Food and Drug Administration
PPIs: Proton pump inhibitors
